# Small molecule-based lineage switch of human adipose-derived stem cells into neural stem cells and functional GABAergic neurons

**DOI:** 10.1038/s41598-017-10394-y

**Published:** 2017-08-31

**Authors:** Jihye Park, Nayeon Lee, Jaekwang Lee, Eun Kyung Choe, Min Kyung Kim, Jeonghoon Lee, Min Soo Byun, Myong-Wuk Chon, Seong Who Kim, C. Justin Lee, Ju Han Kim, Jun Soo Kwon, Mi-Sook Chang

**Affiliations:** 1 Lab. of Stem Cell & Neurobiology, Department of Oral Anatomy, Dental Research Institute and School of Dentistry, Seoul National University, Seoul, 03080 Republic of Korea; 2 Center for Neuroscience and Functional Connectomics, Korea Institute of Science and Technology (KIST), Seoul, 02792 Republic of Korea; 3 Department of Surgery, Seoul National University Hospital Healthcare System Gangnam Center, Seoul, 06236 Republic of Korea; 4 Department of Biochemistry and Molecular Biology, Asan Medical Center, University of Ulsan College of Medicine, Seoul, 05505 Republic of Korea; 5 Division of Biomedical Informatics, Seoul National University Biomedical Informatics (SNUBI), Seoul National University College of Medicine, Seoul, 110799 Republic of Korea; 6 Department of Psychiatry, Seoul National University College of Medicine, Seoul, 03080 Republic of Korea; 7 Department of Psychiatry, Asan Medical Center, Seoul, 05505 Republic of Korea; 8 Department of Brain & Cognitive Sciences, College of Natural Science, Seoul National University, Seoul, 08826 Republic of Korea; 9 Neuroscience Research Institute, Seoul National University, Seoul, 03080 Republic of Korea; 10 Present Address: Division of Functional Food Research, Korea Food Research Institute (KFRI), Seongnam, 13539 Republic of Korea

## Abstract

Cellular reprogramming using small molecules (SMs) without genetic modification provides a promising strategy for generating target cells for cell-based therapy. Human adipose-derived stem cells (hADSCs) are a desirable cell source for clinical application due to their self-renewal capacity, easy obtainability and the lack of safety concerns, such as tumor formation. However, methods to convert hADSCs into neural cells, such as neural stem cells (NSCs), are inefficient, and few if any studies have achieved efficient reprogramming of hADSCs into functional neurons. Here, we developed highly efficient induction protocols to generate NSC-like cells (iNSCs), neuron-like cells (iNs) and GABAergic neuron-like cells (iGNs) from hADSCs via SM-mediated inhibition of SMAD signaling without genetic manipulation. All induced cells adopted morphological, molecular and functional features of their bona fide counterparts. Electrophysiological data demonstrated that iNs and iGNs exhibited electrophysiological properties of neurons and formed neural networks *in vitro*. Microarray analysis further confirmed that iNSCs and iGNs underwent lineage switch toward a neural fate. Together, these studies provide rapid, reproducible and robust protocols for efficient generation of functional iNSCs, iNs and iGNs from hADSCs, which have utility for modeling disease pathophysiology and providing cell-therapy sources of neurological disorders.

## Introduction

Expandable human neural stem cells (NSCs) and functional γ-aminobutyric acid-secreting neurons (GABAergic neurons or interneurons) derived from stem cells are invaluable cell sources for treating nervous system injuries and neurodegenerative diseases. Human embryonic stem cells (ESCs) and induced pluripotent stem cells (iPSCs) can differentiate into all types of cells, making them useful in biomedical research. However, these two stem cells have limitations on clinical application. Besides the ethical concerns in ESCs derivation, iPSCs have safety issues related to exogenous reprogramming factors which may cause the activation of oncogenic and/or unexpected pathways and technical issues about low efficiency with slow reprogramming process. To overcome these limitation, alternative approaches, such as chemicals, microRNAs and direct conversion, have been employed^1–3^. Despite of considerable improvements, there are continued hurdles to get iPSCs safely to the clinic.

Compared to ESCs and/or iPSCs, human adipose-derived mesenchymal stem cells (hADSCs), which represent a type of adult mesenchymal stem cells, are a suitable source for clinical application, because they can be transplanted across allogeneic barriers, reduced ethical concern with obtaining adipose tissue and strong self-renewal capacity^4^. They also can be isolated repeatedly and obtained from patients with an easy and noninvasive procedure^4^. In addition, it has been reported that hADSCs have the neuronal differentiation potential^5^. Moreover, a recent study has reported that hADSCs can be transdifferentiated into NSC- and neuron-like cells, which can generate tetrodotoxin-sensitive sodium current and/or outward potassium current as well as glia-like cells *in vitro* ^6^. Although these results imply the neuronal transdifferentiation capacity of hADSCs, further functional properties, such as action potential or spontaneous postsynaptic current, should be evaluated. However, despite effort to induce hADSCs into neurons or GABAergic neurons, as far as we are aware, there is not an efficient protocol to generate functional neurons, including GABAergic neurons from hADSCs.

Synergistic inhibition of SMAD signaling enhanced neuronal induction efficiency of ESCs and iPSCs by applying small molecules (SMs), the Lefty/Actin/TGFβ pathway inhibitor SB431542 and bone morphogenic protein (BMP) inhibitor noggin together with LDN193189^7^. Several recent studies also demonstrated that fibroblasts could be directly converted into functional human cardiomyocytes and mouse NSCs by SMs^8, 9^. The SMs also promote neural conversion of hADSCs although no electrophysiological properties of induced cells are presented^10^. However, as far as we are aware, there is no published report of generating human NSCs, functional neurons and/or GABAergic neurons from hADSCs by SMs without genetic modification.

Here, we developed an optimal protocol for inducing hADSCs into NSC-like cells (iNSCs) with >85% efficiency using step-wise procedure by SMs without genetic manipulation, which expressed the key NSC markers, such as *Pax6*, *Sox1*, *Nestin* and *Vimentin* ^11^. We also confirmed that the iNSCs were expandable and could maintain their gene expression signatures during serially passaged culture. In addition, we established a highly efficient method for inducing hADSCs into functional neuron-like cells (iNs) within 6 weeks using brain-derived neurotrophic factor (BDNF) and sonic hedgehog (SHH) Smoothened (Smo) activator purmorphamine. Next, we further modified a protocol to generate functional GABAergic neuron-like cells (iGNs) within 7 weeks using BDNF and purmorphamine or cyclic AMP (cAMP) analog dibutyryl-cAMP (dbcAMP). Both the iNs and the iGNs exhibited electrophysiological properties unique to bona fide neurons and GABAergic neurons, respectively, and had gene expression profiles similar to neuronally differentiated human primary NSCs. Taken together, our rapid, reproducible and robust protocols enable the efficient generation of high-quality expandable iNSCs, functional iNs and iGNs from hADSCs, which can be greatly useful for safe and effective cell-based therapy and *in vitro* disease modeling of various neurological and psychiatric disorders.

## Results

### Induction of hADSCs into NSC-like cells using small molecules

**Figure 1:**
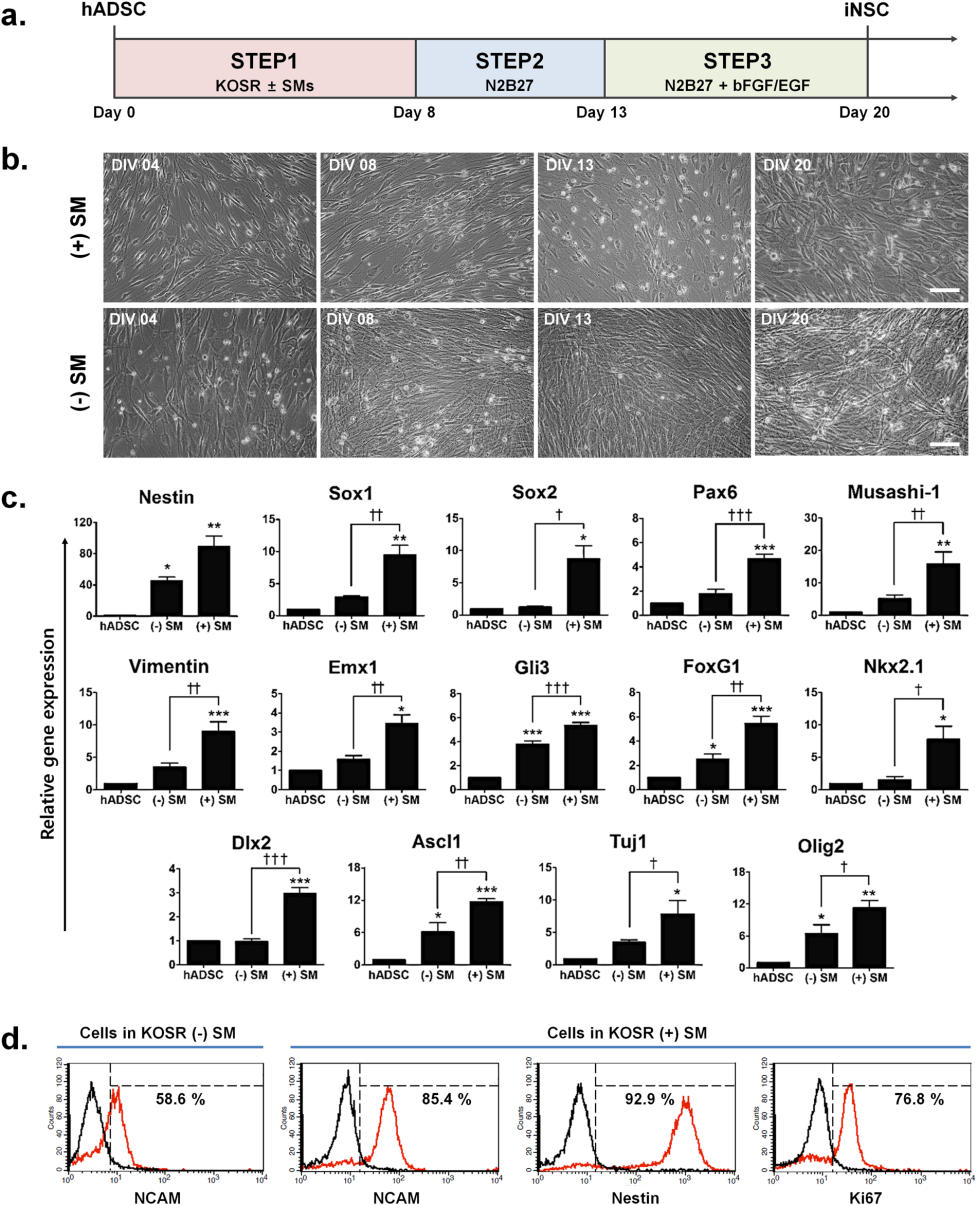
Induction of neural stem cell-like cells (iNSCs) from human adipose-derived mesenchymal stem cells (hADSCs) using small molecules (SMs). **(a)** The experimental scheme for iNSCs derived from hADSCs. **(b)** Bright-field images of hADSCs in culture. Morphological changes were observed at different stages of NSC induction with (+) or without (−) SMs. Scale bar: 100 μm. **(c)** Real-time qPCR of NSC and early neuronal markers, *Nestin*, *Sox1*, *Sox2*, *Pax6, Musashi-1*, *Vimentin, Emx1*, *Gli3*, *FoxG1*, *Nkx2.1*, *Dlx2*, *Ascl1*, *Tuj1* and *Olig2*. The mRNA level of a given gene was quantified by densitometry and normalized to the corresponding *GAPDH* level. The bars represent the mean ± SEM of at least three independent experiments. *P < 0.05, **P < 0.01, ***P < 0.001 compared to hADSC, ^†^P < 0.05, ^††^P < 0.01, ^†††^P < 0.001 compared to (−) SM. ANOVA followed by post hoc Newman-Keuls test. **(d)** Flow cytometry analysis of neural cell adhesion molecule (NCAM)-positive cells after NSC induction with (+) or without (−) SMs as well as Nestin- and Ki67-positive cells after NSC induction with (+) SMs.

hADSCs isolated from three male donors were characterized by flow cytometry to confirm their identity (Supplementary Table S1) and induced into iNSCs using a 3-step NSC induction protocol (Fig. 1a). We determined the lowest concentration of knock out serum replacement (KOSR) required to produce approximately 60% of cells stained positive for neural cell adhesion molecule (NCAM) using flow cytometry analysis (Supplementary Fig. S1). Thus, we used 3% of KOSR for NSC induction of hADSCs for subsequent studies. To further augment neural induction, we supplemented the cultures at STEP 1 with SM inhibitors of TFG-β and BMP signaling pathways; SB431542 (SB), noggin (N) and LDN193189 (L), which are active in early neural development. Real-time quantitative PCR analysis (qPCR) for expressed levels of NSC markers, such as *Sox1*, *Musashi-1* and *Ascl1*, revealed that NSC induction was most efficient when all three compounds were present in the induction media (Supplementary Fig. S2).

Figure 1a illustrates the experimental scheme, which consists of three steps (STEP 1–3), used to confirm the effects of SMs on NSC induction of hADSCs. hADSCs cultured in STEP 1 media with (+) or without (−) SMs exhibited different morphologies (Fig. 1b). Furthermore, after STEP 3, cells in STEP 1 (−) SM media showed significantly higher cell density (Fig. 1b). Real-time qPCR further showed that expression levels of NSC markers associated with early neural development and neural differentiation, such as *Nestin, Sox1*, *Sox2*, *Pax6*, *Musashi-1*, *Vimentin*, *Emx1*, *Gli3*, *FoxG1*, *Ascl1*, *Tuj1* and *Olig2*, were significantly increased in hADSCs cultured in STEP 1 (+) SM media as compared to hADSCs and those in STEP 1 (−) SM media (Fig. 1c). The analysis also revealed that markers of medial ganglionic eminence (MGE)-like ventral forebrain progenitors, such as *Nkx2.1* and *Dlx2*, were significantly increased in hADSCs cultured in STEP 1 (+) SM media as compared to hADSCs and those in STEP 1 (−) SM media (Fig. 1c). Flow cytometry analyses showed that the proportion of NCAM-positive cells in STEP 1 (+) SM media was 85.4% compared to 58.6% in STEP 1 (−) SM media. Furthermore, the proportion of NCAM−, Nestin- and Ki67-positive cells in STEP 1 (+) SM media was 85.4%, 92.9% and 76.8%, respectively, by flow cytometry analysis (Fig. 1d). Taken together, these results suggest that our induction protocol can generate iNSCs from hADSCs with >85% efficiency without the need for genetic manipulation.

### Characterization of iNSCs derived from hADSCs

**Figure 2:**
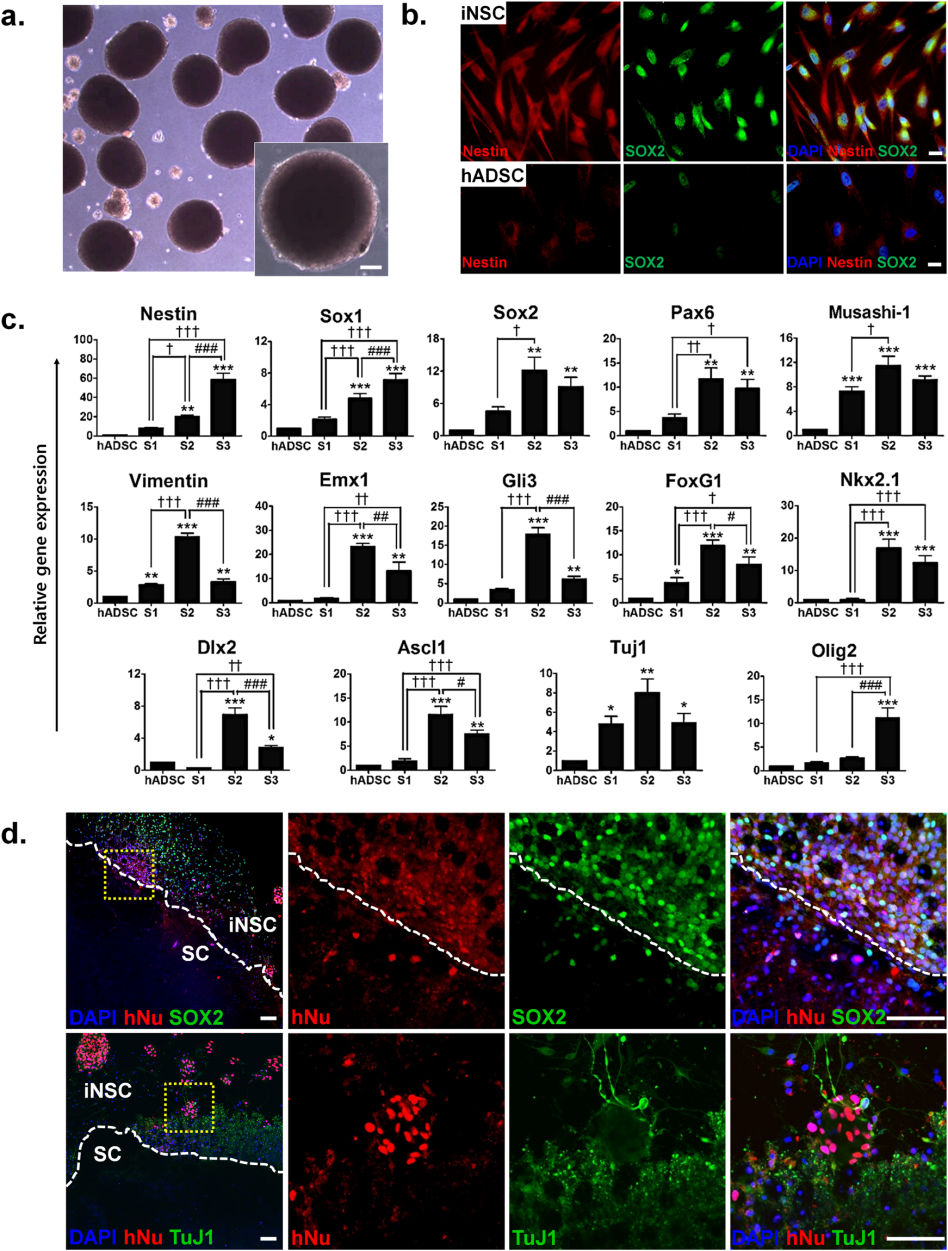
Characterization of neural stem cell-like cells (iNSCs) induced from hADSCs by the optimized induction method. **(a)** Neurosphere formation of iNSCs in suspension at DIV 4 after passage. Scale bar: 100 μm. **(b)** Immunocytochemistry analysis of the expressions of Nestin and SOX2 in iNSCs and hADSCs cultured as a monolayer on cover slips. Scale bar: 20 μm. **(c)** Real-time qPCR of NSC and early neuronal markers, *Nestin*, *Sox1*, *Sox2*, *Pax6*, *Musashi-1*, *Vimentin*, *Emx1*, *Gli3*, *FoxG1*, *Nkx2.1*, *Dlx2*, *Ascl1*, *Tuj1* and *Olig2*, at each step of the induction process. The mRNA level of a given gene was quantified by densitometry and normalized to the corresponding *GAPDH* level. The bars represent the mean ± SEM of at least three independent experiments. *P < 0.05, **P < 0.01, ***P < 0.001 compared to hADSC, ^†^P < 0.05, ^††^P < 0.01, ^†††^P < 0.001 compared to STEP 1, ^#^P < 0.05, ^##^P < 0.01, ^###^P < 0.001 compared to STEP 2. ANOVA followed by post hoc Newman-Keuls test. S1, STEP 1; S2, STEP 2; S3, STEP 3. **(d)** Transplantation of iNSCs onto the ventral horn of rat organotypic spinal cord slices. The transplanted iNSCs were stained with monoclonal anti-human nuclei (hNu, red) and either polyclonal anti-SOX2 or anti-TuJ1 antibodies (green). Scale bar: 100 μm.

As shown in Fig. 2a, iNSCs derived from hADSCs grew as neurospheres in suspension culture, which is a characteristic property of NSCs^12^. It is well-established that bona fide NSCs highly express the intermediate filament Nestin and the transcription factor *Sox2* ^13^. Immunocytochemistry confirmed expression of both Nestin and Sox2 proteins in hADSC-derived iNSCs, whereas expression of these proteins were barely detectable in native hADSCs (Fig. 2b). We further determined the mRNA expression of early neural markers at each phase (STEP 1–3) of the iNSC induction process by qPCR. Expression levels of *Nestin*, *Sox1* and *Olig2* gradually increased throughout the process of iNSC induction (between STEP 1 and 3), whereas *Sox2*, *Pax6*, *Musashi-1*, *Vimentin*, *Emx1*, *Gli3*, *FoxG1*, *Nkx2.1*, *Dlx2*, *Ascl1* and *Tuj1* levels were markedly upregulated at STEP 2 and then subsequently were downregulated during STEP3, such that *Vimentin*, *Emx1*, *Gli3*, *FoxG1*, *Dlx2* and *Ascl1* expressions at STEP 2 were statistically higher than those at STEP 3 (Fig. 2c). To determine whether iNSCs survive and maintain their marker expressions under *ex vivo* culture condition, iNSCs suspended in neurobasal media were transplanted onto the ventral horn of organotypic rat spinal cord slice cultures. One week after transplantation, iNSCs still maintained expressions of SOX2 and TuJ1 under these conditions (Fig. 2d). Taken together, these results suggest that iNSCs acquired morphological and molecular properties of bona fide human NSCs.

### Characterization of neuron-like cells induced from hADSCs

**Figure 3:**
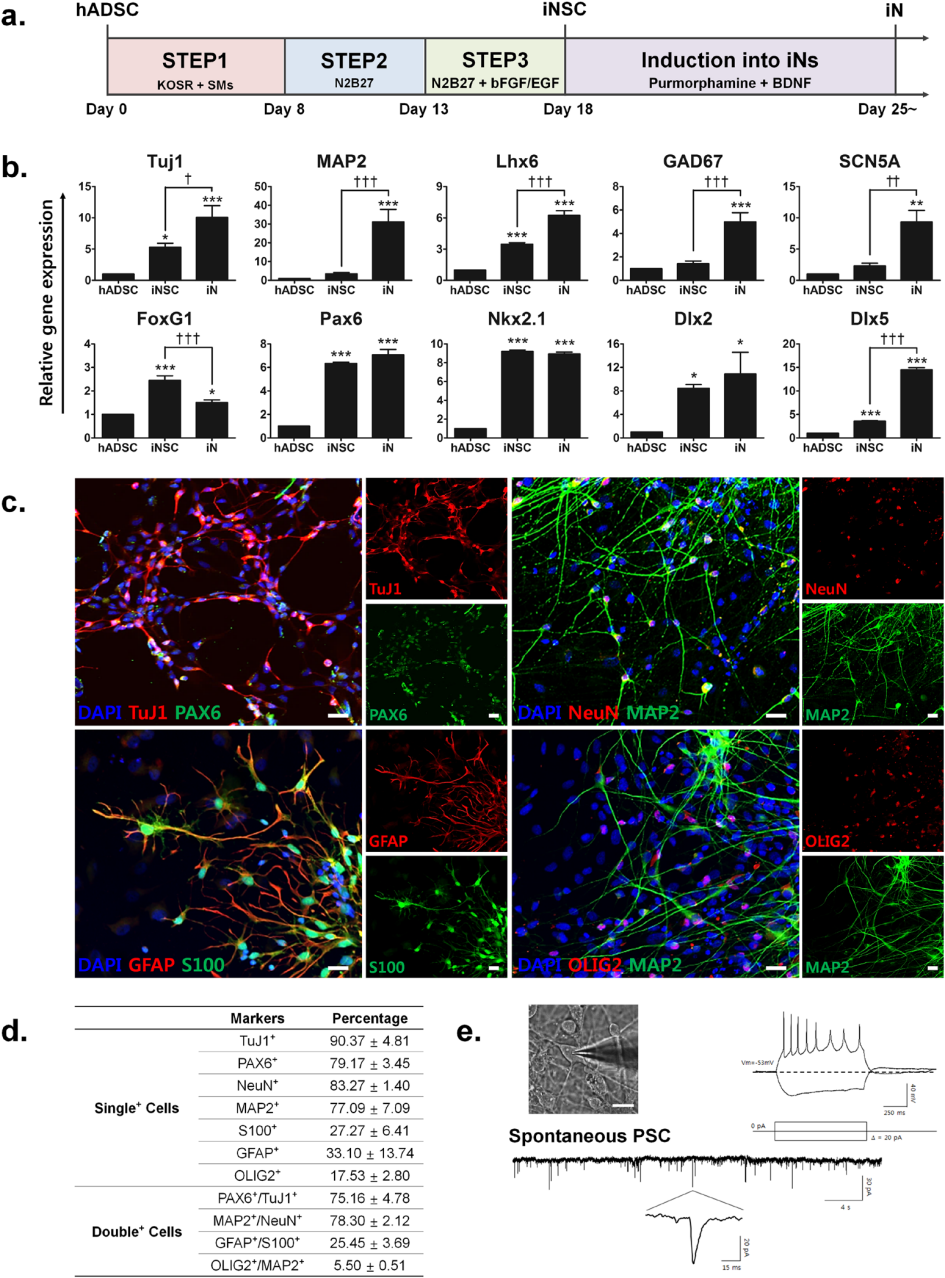
Directed induction of neural stem cell-like cells (iNSCs) into functional neuron-like cells (iNs). **(a)** Experimental scheme for neuronal induction of hADSCs. iNSCs induced from hADSCs were further differentiated into iNs by neuron induction medium containing purmorphamine and BDNF. **(b)** Real-time qPCR of neuronal markers (*Tuj1* and *MAP2*), NSC markers (*FoxG1* and *Pax6*), early and late transcription factors related to medial ganglionic eminence (MGE) (*Nkx2.1*, *Dlx2*, *Dlx5* and *Lhx6*), early GABA marker (*GAD67*) and sodium ion channel (*SCN5A*). The mRNA level of a given gene was quantified by densitometry and normalized to the corresponding *GAPDH* level. The bars represent the mean ± SEM of at least three independent experiments. *P < 0.05, **P < 0.01, ***P < 0.001 compared to hADSC, ^†^P < 0.05, ^††^P < 0.01, ^†††^P < 0.001 compared to iNSC. ANOVA followed by post hoc Newman-Keuls test. **(c)** Neuronal and glial marker protein expressions in iNs; neural precursor markers (TuJ1/Pax6), mature neuron markers (NeuN/MAP2), astrocyte markers (GFAP/S100) and early oligodendrocyte marker (OLIG2). Scale bar: 20 μm. **(d)** Quantitative analysis of neuronal and/or glial marker protein expression in at least three different randomly chosen fields. The percentage represents each marker-positive cells over DAPI-positive cells (mean ± SEM). **(e)** Example of electrophysiological recordings from iNs with typical neuronal morphology. Representative image and induction of action potential (AP) by current injection was shown. The protocol for current injection is below the AP trace. The bottom trace is representative spontaneous synaptic activity obtained from iNs in voltage clamp mode (holding at −60 mV). Magnified single current was shown below the continuous trace.

We next sought to induce hADSCs into mature neurons by further culturing iNSCs in neuronal induction medium containing purmorphamine and BDNF (Fig. 3a). After 7 days of culture in the latter, the cells exhibited neuron-like structures characterized by distinct bipolar or multipolar neurite outgrowth at the periphery of their cell bodies (Supplementary Fig. S3). Real-time qPCR revealed that iNSCs showed increased expressions of *TuJ1*, *MAP2*, *Dlx5*, *Lhx6*, *GAD67* and the sodium ion channel *SCN5A* after 7 day-exposure to neuronal induction media. Interestingly, *FoxG1* was expressed at significantly lower levels in induced neurons (iNs) as compared to iNSCs, *Pax6*, *Nkx2.1* and *Dlx2* mRNA levels were maintained in iNs, demonstrating similar patterns with *in vivo* neuronal development (Fig. 3b).

Immunocytochemistry analysis showed that iNs mainly expressed immature and mature neuronal markers. For example, more than 75% of cells stained double positive for the immature neuronal markers TuJ1 and Pax6, and the mature neuronal markers MAP2 and NeuN (Fig. 3c,d). In contrast, a relatively small fraction (27–33%) of cells expressed astrocyte markers GFAP and S100, indicating the presence of astrocytes at various developmental stages in the culture. Moreover, approximately 17% of cells expressed the oligodendrocyte marker OLIG2, whereas very few cells were double positive for OLIG2 and MAP2 (Fig. 3c,d). iNSCs could be further induced into cells that expressed markers of all three lineages; neurons, astrocytes and oligodendrocytes. However, our data did not unequivocally demonstrate that these cells were iNSCs, since this was not confirmed at the single cell level. Nevertheless, the data suggest that our induction protocol is capable of producing from hADSCs all three cell lineages; mixed neuronal populations and glial cells.

To assess whether iNs exhibit functional properties of neurons, we performed whole-cell patch clamp analysis. In current clamp mode, iNs successfully elicited action potentials (APs) when depolarized by current injection (from −20 to 20 pA). Moreover, some cells showed a depolarizing “sag” in membrane voltage produced in response to injection of hyperpolarizing current. In addition, a spontaneous postsynaptic current (sPSC) was evident in recorded iNs, indicating that these iNs were connected to each other by synapses and formed a neural network (Fig. 3e).

### Optimized induction of hADSCs into GABAergic neuron-like cells (iGNs) and functional characterization of iGNs

**Figure 4:**
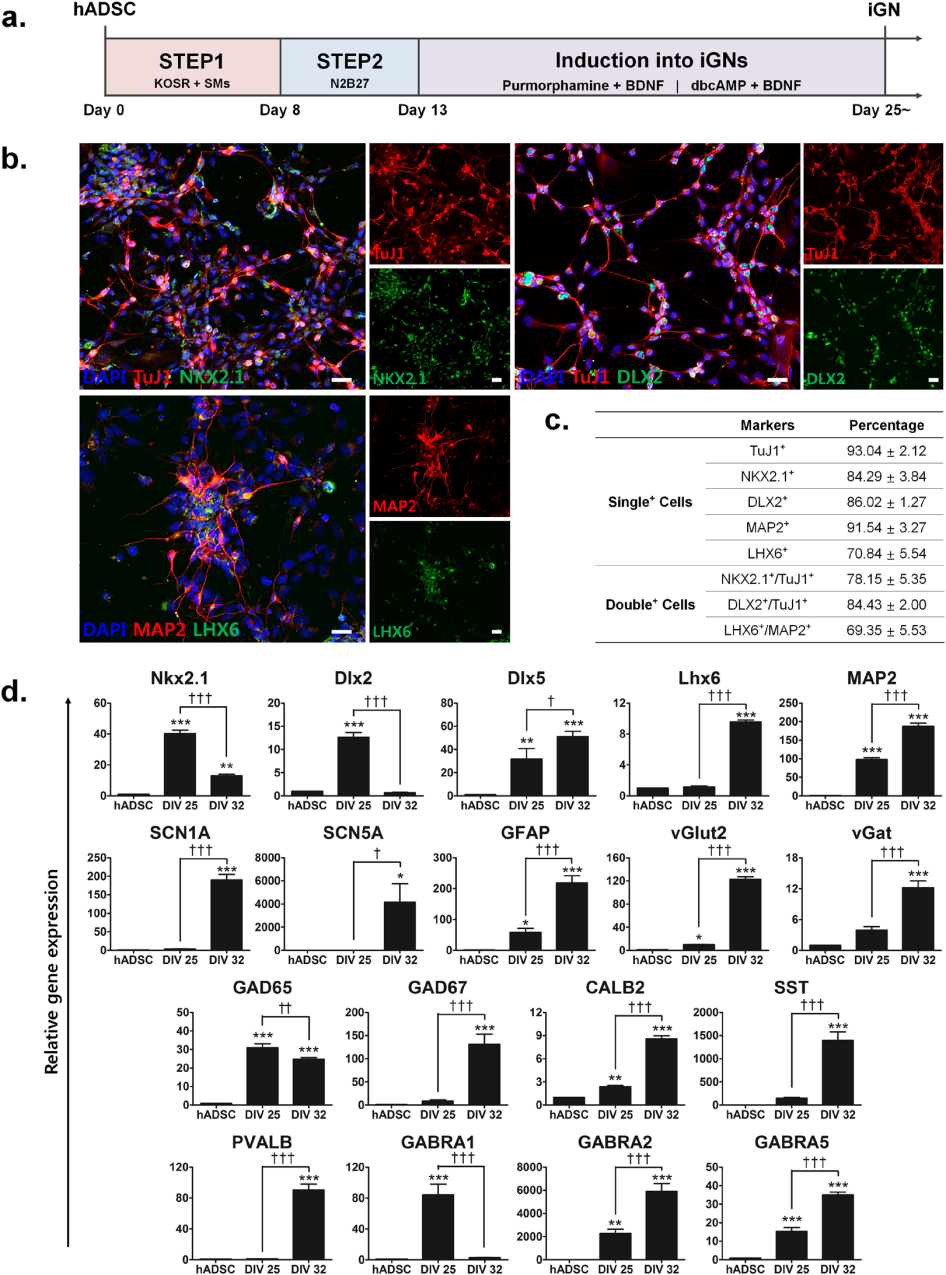
Optimized induction of hADSCs into GABAergic neuron-like cells (iGNs). **(a)** Modified induction scheme of iGNs from hADSCs. STEP 3 of iNSC induction was omitted to expand the days for neuronal maturation and dbcAMP was added to prevent cell death of induced neurons. **(b)** Immunocytochemistry analysis of MGE cell markers NKX2.1, DLX2 and LHX6 with neuron markers TuJ1 and MAP2 in iGNs. Scale bar: 20 μm. **(c)** Quantitative analysis of MGE cell and/or neuron marker protein expression in at least three different randomly chosen fields. The percentage represents each marker-positive cells over DAPI-positive cells (mean ± SEM). **(d)** Real-time qPCR of MGE transcription factor (*Nkx2.1*, *Dlx2*, *Dlx5*, *Lhx6*), neuron marker (*MAP2*), sodium ion channel (*SCN1A*, *SCN5A*), astrocyte (*GFAP*), glutamatergic neuron marker (*vGlut2*), GABAergic neuron marker (*vGat*, *GAD65*, *GAD67*), GABAergic interneuron marker (*CALB2, SST, PVALB*) and GABA receptor (*GABRA1, GABRA* and *GABRA5*). The further induced iGNs (DIV 32) mostly showed dramatically increased gene expression levels of mature GABAergic neurons but decreased expression of relatively early GABAergic neuron markers, compared to iGNs at DIV 25. The mRNA level of a given gene was quantified by densitometry and normalized to the corresponding *GAPDH* level. The bars represent the mean ± SEM of at least three independent experiments. *P < 0.05, **P < 0.01, ***P < 0.001 compared to hADSC, ^†^P < 0.05, ^††^P < 0.01, ^†††^P < 0.001 compared to DIV 25. ANOVA followed by post hoc Newman-Keuls test.

As described above (Fig. 2), iNSCs at STEP 2 expressed high levels of NSC-specific markers, such as Nestin and Sox2. In addition, the levels of several transcription factors important for GABAergic neuronal differentiation, including *Dlx2* and *Ascl1*, were substantially decreased at STEP 3. Thus, we decided to use iNSCs at STEP 2 for GABAergic neuronal induction and added dbcAMP into the culture to prevent cell death of induced neurons (Fig. 4a). At days *in vitro* (DIV) 25, the majority of cells expressing neural markers TuJ1 or MAP2 were also positive for NKX2.1 (approx. 78%), DLX2 (approx. 84%) and LHX6 (approx. 69%), specific markers for MGE originating from the ventral telencephalon ventricular zone (Fig. 4b,c).

To further investigate the GABAergic neuronal properties of iGNs, real-time qPCR was performed to evaluate the mRNA levels of various GABAergic neuronal markers at DIV 25 and DIV 32. The level of *MAP2*, a marker of mature neurons, increased substantially at DIV 25 compared to native hADSCs (P < 0.001) and further increased at DIV 32 (Fig. 4d). Similarly, the expression of the sodium ion channel subtype markers *SCN1A* and *SCN5A* significantly increased in both hADSCs and iGNs at DIV 32 as compared to DIV 25 (Fig. 4d). In addition, while iGNs matured from DIV 25 to DIV 32, the early and late MGE transcription factor *Nkx2.1*, *Dlx2*, *Dlx5* and *Lhx6* respectively, displayed an expression pattern consistent with that observed during MGE progenitor differentiation into GABAergic neurons *in vivo* (Fig. 4d). Interestingly, in addition to vesicular GABA transporter *vGat*, the expression of the vesicular glutamate transporter *vGlut2* and astrocyte intermediate filament *GFAP* significantly increased in both hADSCs and iGNs at DIV 32 compared to DIV 25, suggesting that some iNSCs were also induced into glutamatergic neuron-like cells and astrocyte-like cells in the culture (Fig. 4d).

For iGNs to exhibit functional properties of GABAergic neurons, it is essential to have GABA release from the presynaptic neurons and expression of GABA receptors on postsynaptic neurons. GABA_A_ receptor, one of two classes of GABA receptors, is an ionotropic receptor consisting of various types of subunits, including GABRA1, GABRA2 and GABRA5. The significant increase of *GABRA2* and *GABRA5* mRNA levels was continuous until DIV 32 (Fig. 4d). However, the significantly increased *GABRA1* mRNA level at DIV 25 decreased up to the level similar to controls. Glutamic acid decarboxylase (GAD) is an enzyme that converts glutamate to GABA, existing in two isoforms GAD65 and GAD67. GAD65 and GAD67 synthesize GABA for functionally different purpose; GAD65 for neurotransmission and GAD67 for neuronal activity. The mRNA level of *GAD65* was significantly enhanced at DIV 25 and sustained at DIV 32 compared to native hADSCs (Fig. 4d). However, the mRNA level of *GAD67* was not increased until DIV 32, indicating that the neuronal activity, such as synaptogenesis, exceedingly occurred during neuronal maturation (Fig. 4d). GABAergic cortical interneurons can be distinguished into several subgroups based on the molecular markers, including calbindin2 (CALB2); also known as calretinin (CR), somatostatin (SST) and parvalbumin (PV). In addition, MGE progenitors mostly differentiate into SST− and PV−expressing interneurons^14^. Consistent with this developmental pattern, the mRNA expression of *CALB2*, *SST* and *PVALB* significantly increased in iGNs at DIV 32 compared to both hADSCs and iGNs at DIV 25, indicating that our protocol can generate iGNs with these GABAergic interneuron subtypes. These results suggest that iGNs may adopt the feature of MGE-derived inhibitory GABAergic neurons.

**Figure 5:**
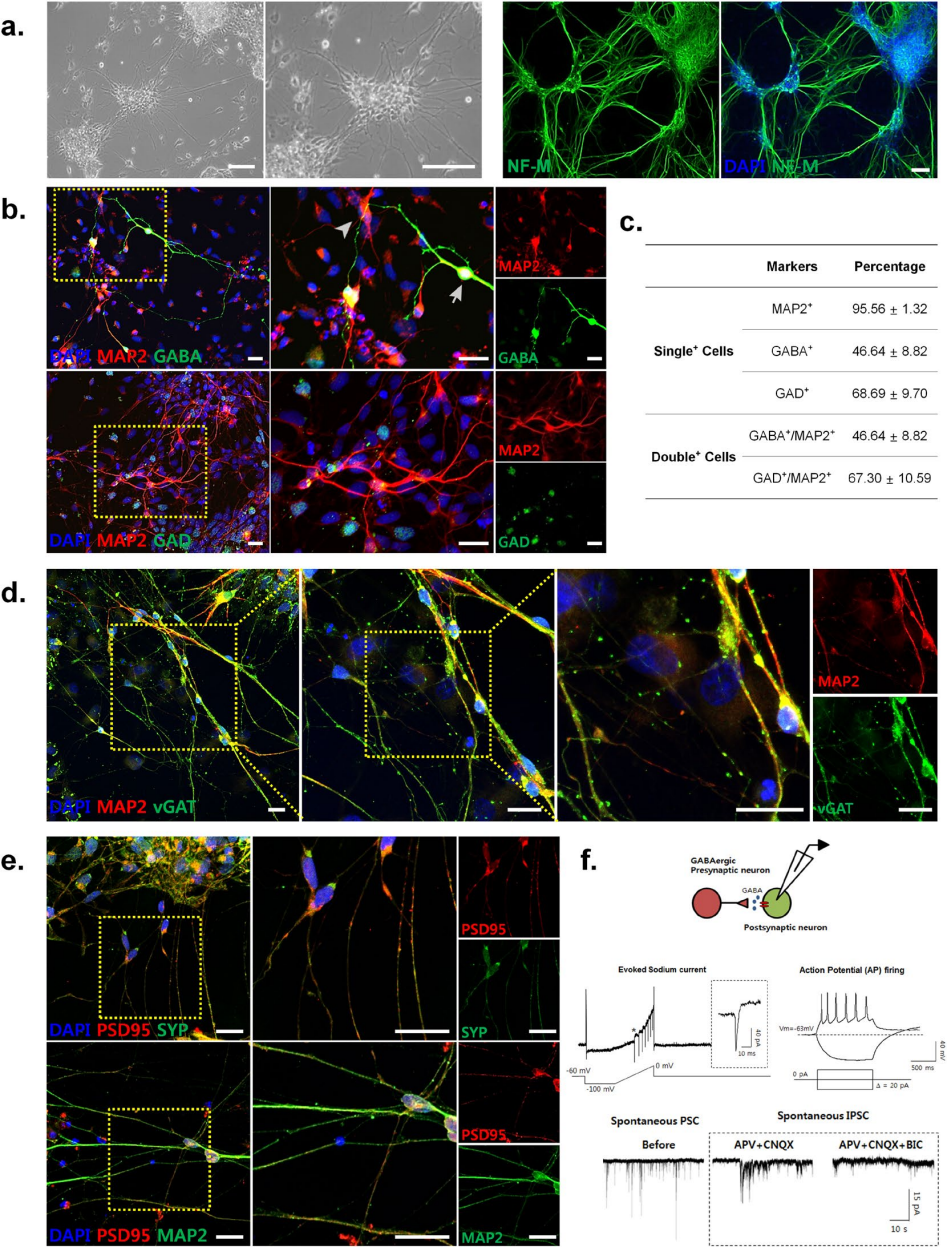
Immunocharacterization and electrophysiological properties of GABAergic neuron-like cells (iGNs) induced from hADSCs. **(a)** Neurofilament-M (NF-M)-immunoreactive neurites radiating from the neurosphere-like cell cluster. Scale bar: 100 μm. **(b)** Immunocytochemistry analysis of mature neuron (MAP2) and GABAergic neuron (GABA, GAD) expressions. Scale bar: 20 μm. **(c)** Quantitative analysis of GABAergic neuron and/or mature neuron marker protein expression in at least three different randomly chosen fields. The percentage represents each marker-positive cells over DAPI-positive cells, the total cell number (mean ± SEM). **(d)** Immunocytochemistry analysis of MAP2 and pre-synaptic marker of GABAergic neuron (vGAT) expressions. Scale bar: 20 μm. **(e)** Immunocytochemistry analysis of MAP2 and pre-/post-synaptic marker (PSD95/SYP) expressions. Scale bar: 20 μm. **(f)** Representative trace of AP firing from iGNs recorded in current clamp mode (upper left panel). The protocol for current injection is below the AP trace. The upper right panel shows that applying ramp protocol in iGNs elicited fast inward current followed by depolarization of the holding voltage. Voltage was ramped from −100 to 0 mV gradually for 1 sec. The dotted box indicates magnification of ramp current marked with a star (*). A schematic diagram of the experimental design is shown in the bottom left panel. Spontaneous inhibitory postsynaptic current (IPSC) in the company of glutamatergic receptor blockers (50 M APV and 20 M CNQX) is shown in the bottom right and was not observed in the presence of 10 M bicuculine, a GABA_A_ receptor blocker.

We also observed NF-M-positive elongated neurites radiating from the neurosphere-like cell cluster and extended neurite outgrowth forming neural networks in the culture (Fig. 5a). Consistent with these data, immunostaining revealed that many MAP2-positive iGNs expressed GABAergic neuron-specific markers GABA (approx. 47%) and GAD (approx. 67%) (Fig. 5b,c). In addition, some MAP2-positive iGNs were labeled with the presynaptic inhibitory synapse marker vGAT (Fig. 5d). MAP2-positive iGNs were also immunopositive for the postsynaptic excitatory synapse marker PSD95, which is co-labeled with the synaptic protein synaptophysin (SYP). Both PSD95 and SYP exhibited punctuated staining indicative of possible synaptic connections (Fig. 5e).

In order to verify functional synaptic formation, electrophysiological properties of iGNs were examined at DIV 32. Before measuring inhibitory postsynaptic current (IPSC), intrinsic electrical properties were measured by eliciting APs. The cells exhibited a fast Na^2+^ inward current during depolarization of membrane potential. Next, IPSC was measured in the presence of D-APV (50 μM) and 6-cyano-7-nitroquinoxaline-2,3-dione (CNQX, 10 μM), NMDAR antagonist and AMPAR antagonist, respectively. The presence of IPSCs was frequently observed and the isolated IPSCs were further blocked by bicuculline (BIC, 10 μM), indicating that iGNs exhibited electrophysiological features of functional GABAergic neurons (Fig. 5f).

### Microarray analysis of iNSCs and iGNs induced from hADSCs

**Figure 6:**
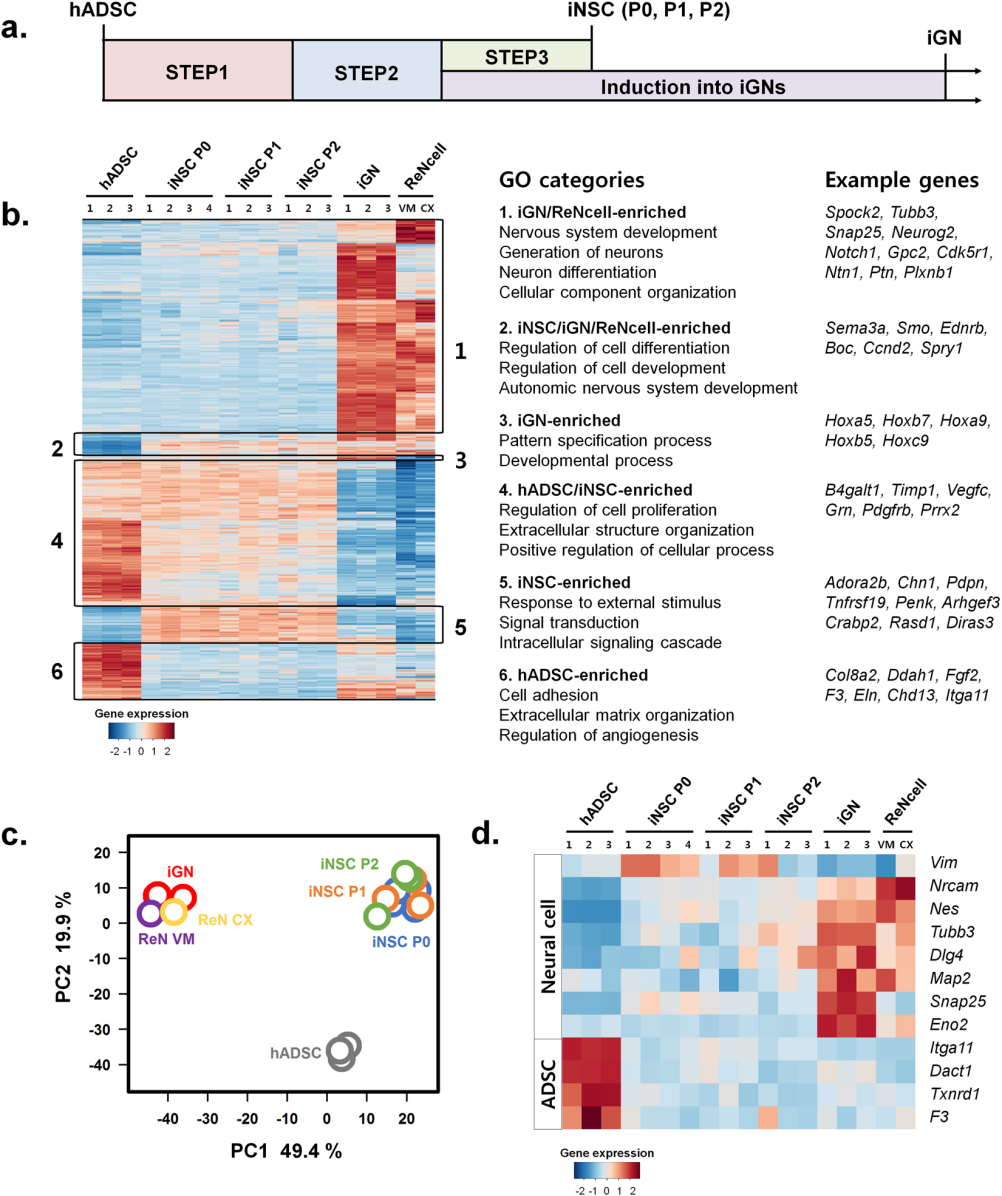
Microarray analysis of neural stem cell-like cells (iNSCs) and GABAergic neuron-like cells (iGNs) induced from hADSCs. **(a**) Schematics of samples for microarray analysis. The passage numbers of iNSCs are presented as iNSC P0, P1 and P2. **(b)** Heatmap for differentially expressed genes (P < 0.005, Kruskall-Wallis test with Conover correction for multiple comparisons). The numbers above heatmap depict independent biological replicates of each group. The neuronally differentiated ReN VM and CX cells were used as positive controls of human neurons. The right panel lists Gene Ontology (GO) annotations and example genes for each cluster. **(c)** Multidimensional scaling of hADSCs, iNSCs, iGNs, ReN VM and CX samples. **(d)** Heatmap representation of the expression profiles of 8 neural cell- and 4 hADSC-enriched genes at each sample. The numbers above heatmap depict independent biological replicates of each group.

To further validate the induction protocol, gene expression profiles of cells at different time points during the induction process were analyzed by microarray analysis (Fig. 6a). Furthermore, to examine the stability of iNSC phenotype, cells at different passage numbers (P0, P1 and P2) were included in microarray analysis. ReN VM and CX cells induced to mature neural phenotypes were used as positive controls of human neurons.

Gene Ontology (GO) analysis revealed that genes involved in biological processes of nervous system development, generation of neurons and neuron differentiation were highly expressed in both iGNs and differentiated ReNcells, indicating that iGNs acquired the gene expression signatures of mature neurons (Fig. 6b). During induction of hADSCs into iNSCs and iGNs, genes associated with regulation of cell differentiation/development were upregulated, whereas genes involved in hADSC functions, such as cell adhesion and extracellular matrix organization, were downregulated (Fig. 6b). Principle component analysis showed that iGNs/differentiated ReNcells, iNSCs P0/P1/P2, and hADSCs were clustered and each cluster was well-distinct with one another (Fig. 6c). In addition, the heatmap analysis of 8 neural cell- and 4 hADSC-enriched gene expressions demonstrated that neural genes were upregulated, but hADSC genes were significantly downregulated in iNSCs, iGNs and ReNcells (Fig. 6d). Furthermore, we performed REACTOME pathway-enrichment analysis and identified REACT_13685:Synaptic Transmission as a statistically significant pathway related to the differentially expressed genes in iGNs. Taken together, these results indicate the efficient cellular reprogramming of hADSCs into neural cells.

## Discussion

Our study demonstrates for the first time that hADSCs without genetic manipulation can be induced into expandable iNSCs with high efficiency, 85.4% of NCAM^+^ cells. This occurred with SM inhibitors of Lefty/Actin/TGFβ and BMP pathways. Additionally, hADSCs can be induced into functional iNs and/or iGNs with a neuronal purity greater than 75% and 46%, respectively.

**Figure 7:**
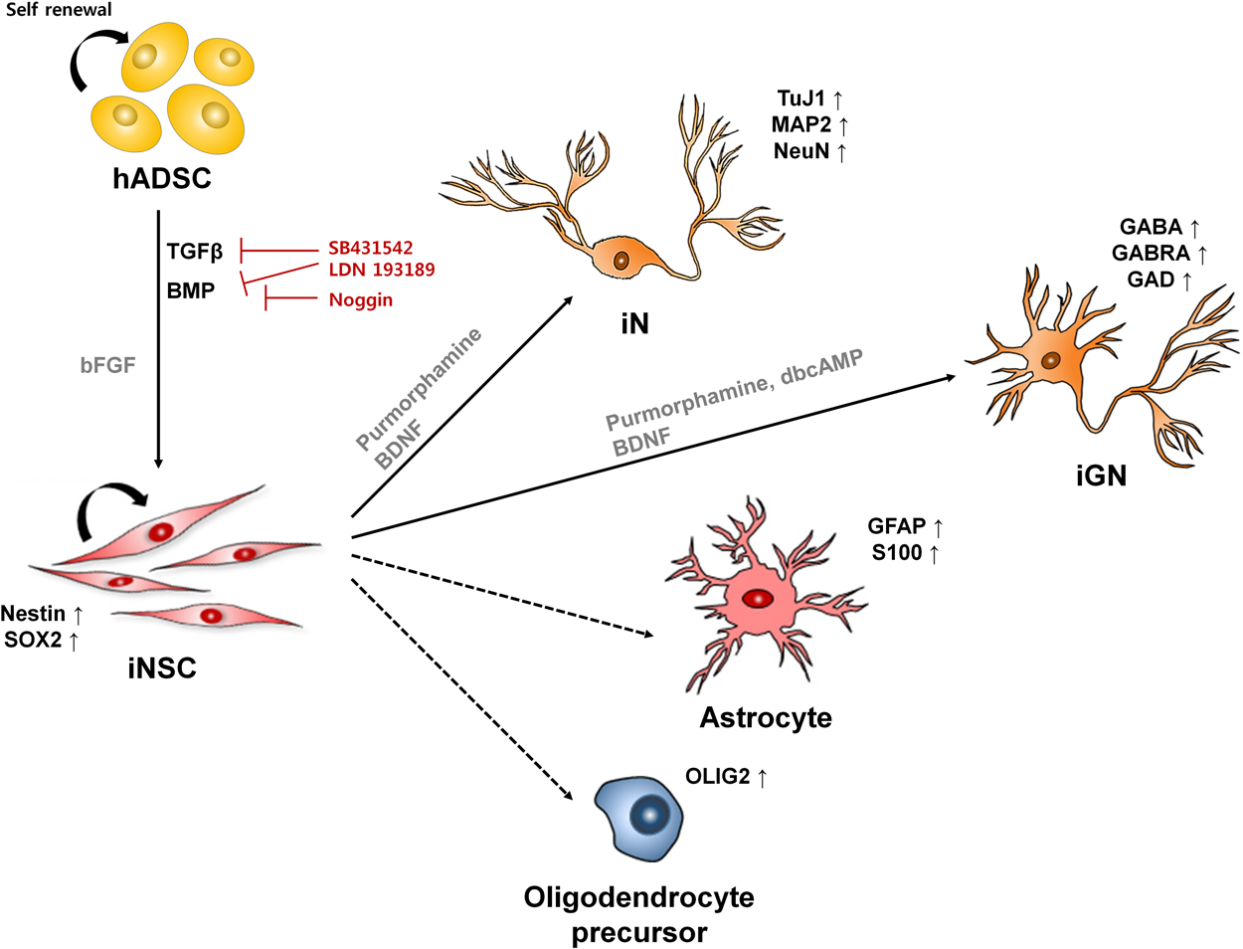
Graphical summary of inducing hADSCs into neural stem cell-like cells (iNSCs), neuron-like cells (iNs) and GABAergic neuron-like cells (iGNs). Our optimized induction protocol using an SM combination efficiently induces hADSCs into iNSCs, which have self-renewal capacity. iNSCs can also be induced into iNs and iGNs, and possibly into astrocytes and oligodendrocyte precursors.

iNSCs could self-renew and form neurospheres in suspension culture as bona fide NSCs. Moreover, iNSCs exhibited features of MGE-like ventral forebrain progenitors, e.g. they exhibited significantly increased expression of *Nkx2.1* and *Dlx2* compared to native hADSCs. These MGE-like progenitors were further induced into mature neuron-like cells. We also observed cells expressing glial markers, such as GFAP, S100 and OLIG2, indicating that iNSCs could be further induced into neurons, astrocytes and oligodendrocytes (Fig. 7). Due to their self-renewal capacity, iNSCs can be cryopreserved and may be used as a stable intermediate for the generation of neurons and glia. In our study, iNSCs can maintain their gene expression signatures during serially passaged culture and after cryopreservation. In addition, iNSCs can be transplanted *ex vivo* with good viability making them suitable for clinical applications. For example, we showed that iNSCs transplanted into spinal cord slice cultures maintained expressions of SOX2 and TuJ1 up to one week post-transplantation (Fig. 2d). Therefore, iNSCs may represent an alternative cellular therapeutic for neurodegenerative diseases, although further evaluation is needed using appropriate translational models to assess their potential efficacy.

The potency of iNSCs was evaluated by differentiating into mature neurons with purmorphamine targeting Smo to activate the SHH pathway and BDNF^15, 16^. Although it has been reported that sole treatment with BDNF can induce hMSCs into neuron-like cells^5, 17^, as far as we are aware, there is no report of the use of both purmorphamine and BDNF to induce hADSCs into neurons. Here, we developed an efficient protocol for neuronal induction of hADSCs by activating both SHH and BDNF pathways. In addition to morphological and molecular features of neurons, iNs exhibit excitable properties of neurons and sPSC, indicating possible synaptic networks in the culture (Fig. 3e).

Importantly, our study demonstrates for the first time that functional iGNs can be induced from hADSCs without genetic modification, such as transfection of proneural transcription factors. Although iN is a useful source for the research of molecular and/or developmental mechanisms of neurodegenerative disorders, it is hardly applicable to neurological pathologies caused by impaired excitation and inhibition (E/I) balance in neuronal networks. Dysfunction of GABAergic neurons is involved in many neurological and psychiatric disorders due to their crucial role in E/I balance in the brain^18, 19^. Quite a few studies have reported that GABAergic interneuron transplantation exhibited therapeutic effects in animal models of neurological disorders, such as epilepsy and Parkinson’s disease, by modifying neural circuits, suggesting a possible application of cell-based therapy for human neurological and neurodegenerative disorders^19^. Also, iGNs with specific pathological traits can facilitate studies of pathogenesis and *in vitro* disease modeling.

A recent study demonstrates that human fibroblasts can be converted into functional GABAergic neurons by introducing five factors (*FoxG1*, *Sox2*, *Ascl1*, *Dlx5* and *Lhx6*)^20^. In our study, the mRNA levels of *FoxG1*, *Sox2*, *Dlx2* and *Ascl1* at STEP 2 of iNSC induction were significantly upregulated compared to those in hADSCs and even higher than those at STEP 3. In addition, iNs expressed significantly higher mRNA levels of cortical interneuron transcription factors *Dlx5* and *Lhx6* and early GABA transcription factor *GAD67* compared to native hADSCs and iNSCs, indicating that iNs can be possibly further differentiated into GABAergic neurons. Thus, we selected STEP 2 cells to optimize the protocol for GABAergic neuronal induction by step-wise procedures: neuronal induction medium consisting of purmorphamine and BDNF, and further differentiation with dbcAMP and BDNF. Many MAP2-positive iGNs expressed GABA (approx. 47%) and GAD (approx. 67%), and some MAP2-positive iGNs were immunopositive for vGAT, PSD95 and SYP, indicating possible inhibitory and excitatory synaptic network formation *in vitro*. Microarray analysis also demonstrated that iGNs acquire gene expression signatures of mature neurons and highly express genes involved in synaptic transmission. Consistently, whole-cell patch clamp recordings show that iGNs generate evoked sodium currents together with action potentials and spontaneous IPSCs, confirming the existence of functional GABAergic neurons. Our simple, rapid and robust protocol enables us to generate functional iGNs from hADSCs and requires even less time than the iGN-induction protocol using hiPSCs in a recent study^3^. Although our results illustrate the development of an optimal method for generating functional iGNs from hADSCs without genetic manipulation, further studies are needed to fully characterize the functional attributes of iGNs as well as their therapeutic potential, if any.

A recent study has demonstrated that a cocktail of nine SMs can convert mouse fibroblasts into mouse NSCs, suggesting the feasibility of generating human NSCs and neurons using SMs only^9^. Moreover, the SMs also promote differentiation of hADSCs into neuron-like cells expressing *Sox1*, *Pax6* and *NF-H* ^10^. SMs are ideal for generating target cells similar to human bona fide *in vivo* counterparts because they are safe, stable and cost-effective. Also, SMs are easy to manipulate and allow precise temporal control of conversion. In addition, SMs provide insights on the underlying mechanisms of cell conversion by targeting specific pathways. However, especially for neural induction, relatively low efficiency of chemical induction has limited broad application compared to induction with genetic manipulation, such as forced expression of proneural genes. However, introducing exogenous genes may cause variability of target cells depending on transfection efficiency and safety concerns, such as activation of unexpected pathways. Thus, it is safer and more stable to generate neural cells by activating endogenous neural genes using SMs.

However, as far as we are aware, there is no published report of the generation of human NSCs and/or functional GABAergic neurons from hADSCs using SMs without genetic modification. Here, for the first time, we developed an efficient protocol for inducing hADSCs into iNSCs, iNs and iGNs through synergistic inhibition of the SMAD signaling pathway by applying three SMs, SB431542, noggin and LDN193189. The SMs promoted mRNA expression of genes related to neural development, such as *Sox1*, *Pax6* and *Musashi-1* (Fig. 1c). The neural tube, the primordium of the central nervous system, consists of neuroepithelial cells expressing Pax6 and Sox1, which are crucial factors in neural cell fate determination and/or differentiation^21^. In addition, the *Musashi-1* is selectively expressed in mammalian NSCs, and plays an important role in maintenance of NSC state and differentiation^22^. Dual inhibition of SMAD signaling during iNSC induction also upregulates genes associated with NSCs and/or neural progenitors, such as *Vimentin*, *FoxG1*, *Ascl1* and *Olig2*, which leads to enhanced *Tuj1*, a neuron-specific microtubule expressed in immature neurons^23^. Taken together, our results demonstrate efficient and reproducible generation of functional iNSCs, iNs and iGNs using only SMs without genetic manipulation of hADSCs, which are known to be refractory to neural conversion.

## Methods

### Isolation and culture of hADSCs

Adipose tissue was obtained through liposuction^24^ from the healthy volunteers (23–26 years old) provided with informed consent. All procedures were performed in accordance with the Declaration of Helsinki and were approved by the Institutional Review Board of Seoul National University Hospital. Isolated hADSCs were maintained in culture medium consisting of alpha-minimum essential medium (α-MEM; Hyclone) supplemented with 10% fetal bovine serum (Hyclone) and 100 μg/ml penicillin and streptomycin (Gibco)^25﻿^. Cells were subcultured every 3–4 days at a density of 1,400 cells/cm^2^ and maintained at 37 °C with 5% CO_2_. hADSCs at passage 4 were used for all experiments.

### Induction of hADSCs into iNSCs

hADSCs were seeded on gelatin-coated dishes (10,000 cells/cm^2^) in NSC induction medium consisting of Dulbecco’s modified Eagle’s medium F12 (DMEM/F12; Gibco) supplement with 3% KOSR (Gibco), 1% Glutamax (Gibco), 1% non-essential amino acid (Gibco) and 4 ng/mL basic fibroblast growth factor (bFGF; Peprotech) with or without three small molecules; 10 μM SB431542 (Sigma), 100 ng/ml noggin (R&D) and 0.5 μM LDN193289 (Stemgent), for 8 days (STEP1). Next, cells were cultured in neurobasal medium (Gibco):DMEM/F12(1:1) supplemented with 2% B27 (Gibco), 1% N2 (Gibco), 1% Glutamax (Gibco) and 200 μM ascorbic acid (Sigma) for 5 days (STEP2). The medium was replaced with STEP2 medium supplemented with 20 ng/mL epidermal growth factor (EGF; Peprotech) and 20 ng/mL bFGF for another 7 days (STEP3).

### Induction of iNSCs into iNs and iGNs

iNSCs were dissociated using TryPLE select (Gibco) and replated (50,000 cells/cm^2^) on plastic cover slips (Thermanox; Thermo) coated with 5 μg/ml poly-L-ornithine (PLO; Sigma) and 50 μg/ml fibronectin (FN; Sigma). On the next day, the medium was replaced with neural induction medium; STEP2 medium containing 1 μM purmorphamine (Tocris) and 10 ng/ml brain-derived neurotrophic factor (BDNF; R&D) for 12–14 days.

For GABAergic neuronal induction, the cells at STEP2 were incubated with the neural induction medium for 12–14 days, followed by STEP2 medium consisting of 50 μM dbcAMP (Sigma) and 20 ng/ml BDNF for another 12–14 days.

### Culture and neuronal differentiation of ReNcell VM and CX

Two immortalized stem cell lines, ReNcell-VM from midbrain (Millipore) and -CX from cortex (Millipore), were cultured on 1 μg/ml PLO and 10 μg/ml FN-coated dishes in growth medium of DMEM/F12 supplemented with 1% N2, 1% Glutamax, 20 ng/mL EGF and 20 ng/mL bFGF. Subculture was performed every 3–4 days using Accutase (Gibco) and reattached (50,000 cells/cm^2^).

For neuronal differentiation, cells (passage 20–30) were seeded in dishes (50,000 cells/cm^2^) for spontaneous aggregation and expanded in growth medium for 5–7 days. Aggregates were replated on PLO/FN-coated plates and then differentiated for 10 days using growth medium containing 1 mM dbcAMP and 2 ng/ml GDNF (Peprotech)^26^.

### Quantitative real-time PCR

Total RNA was extracted using the TRIzol® reagent (Invitrogen). After DNase treatment, cDNA was synthesized by M-MLV reverse transcriptase (Promega) at 42 °C for 1 h. Quantification of genes was performed using SYBR FAST qPCR Kits (KAPA biosystems). PCR amplification was generated using gene-specific primers (Supplementary Table S2). The target gene expression was normalized to endogenous *GAPDH*, and the level of genes was determined by the comparative *C*t method. The *C*t value is the cycle number at which the fluorescence level reaches threshold. The *ΔC*t value is determined by subtracting the *C*t value of the *GAPDH* control from the *C*t value of the target gene [*ΔC*t = *C*t(Target) − *C*t(*GAPDH*)]. This relative value of target genes to endogenous reference is described as the fold-change of *GAPDH* = 2^−*ΔC*t^.

### Immunocytochemistry

Cells were fixed with 4% paraformaldehyde (PFA) in PBS, permeabilized and blocked with 0.1% Triton-X100 plus and 5% normal goat serum in PBS. Then, they were incubated overnight at 4°C with the following primary antibodies: mouse anti-DLX2, 1:500 (Santa Cruz), rabbit anti-GABA, 1:1000 (Sigma), rabbit anti-GAD65/67, 1:250 (Millipore), mouse anti-GFAP, 1:200 (Millipore), mouse anti-MAP2, 1:200 (Chemicon), rabbit anti-MAP2, 1:1000 (Millipore), mouse anti-nestin, 1:200 (Chemicon), mouse anti-NeuN, 1:500 (Millipore), rabbit anti-neurofilament M, 1:200 (Millipore), mouse anti-NKX2.1, 1:1000 (Millipore), rabbit anti-OLIG2, 1:1000 (a gift from Dr. Charles Stiles, Harvard Medical School), mouse anti-PAX6, 1:250 (Millipore), mouse anti-PSD95, 1:500 (Millipore), rabbit anti-S100, 1:250 (Dako), mouse anti-SOX2, 1:500 (Millipore), rabbit anti-synaptophysin, 1:250 (Sigma), rabbit anti-TuJ1, 1:1000 (BioLegend) and mouse anti-vGAT, 1:200 (Synaptic Systems). After washing with PBS, cells were incubated with the secondary antibodies, Alexa Fluor® 555 anti-mouse IgG (Molecular Probes) and Alexa Fluor® 488 anti-mouse IgG (Molecular Probes). Cells were then counter-stained with 4,6-diamidino-2-phenylindole (DAPI) (Santa Cruz). The images were captured using a confocal laser-scanning microscope (LSM700; Zeiss) and digital inverted fluorescence microscope (DM5000B; Leica).

### Fluorescence-activated cell sorter (FACS) analysis

hADSCs were incubated with each antibody against human CD14, CD34, CD45, HLA-DR (FITC; BD), CD44, CD73, CD90, CD105, CD166 (PE; BD) and CCR2 (Alexa Fluor; Serotec) for 1 h at 4 °C. Corresponding mouse isotype antibodies were used as controls. The single color-stained cells were washed with PBS and fixed with 1% PFA in PBS. MSC immunotypes were determined by flow cytometry on a FACS Calibur System (BD) and the percentage of expressed cell surface antigens was calculated for 10,000 gated-cell events.

To quantify the percentage of iNSCs induced from hADSCs, iNSCs were incubated with NCAM-conjugated FITC, Nestin-conjugated PE (BD) and Ki67-conjugated FITC (eBioscience) for 1 h at 4 °C. Cells without antibody binding were used as controls. FACS experiment and data analysis were carried out according to manufacturer's instructions (FACS Calibur System, BD).

### Slice culture and cell transplantation analysis

All procedures were approved by the Institutional Animal Care and Use Committee of Seoul National University. The organotypic slice culture was performed as previously described^27–29^. Cells (6 × 10^3^ cells/μl) prepared in neurobasal medium were transplanted onto the slice using aspirator tube assembly for microcapillary pipette (Sigma). In 7 days, the slices were fixed with 4% PFA overnight at 4°C, permeabilized and blocked with 0.1% Triton X-100 in 3% BSA. They were incubated overnight at 4°C with mouse anti-SOX2, 1:500, rabbit anti-TuJ1, 1:1000 and mouse anti-human nuclei, 1:500 (Millipore). Incubation with the secondary antibodies was performed as described above. The z-stack images were captured using a laser-scanning confocal microscope at 3–4 µm intervals.

### Electrophysiology

AP firing and spontaneous post synaptic current were recorded from cultured iN at DIV 22–32 days. The cover slip was placed in recording solution containing (in mM) 150 NaCl, 10 HEPES, 3 KCl, 2 CaCl_2_, 2 MgCl_2_, 5.5 glucose and 20 sucrose with pH 7.3. The recorded cell was visualized either directly via the microscope’s optics, or indirectly via a high-resolution CCD camera system (Orca Flash 2.1, Hamamatsu) that received the output of a CCD camera attached to the microscope’s video port. Whole-cell patch clamp recordings were obtained using borosilicate glass pipettes (resistance 4–8 MΩ) prepared using a 2-stage vertical pipette puller (Narishige PC-10). Voltage and current clamp recordings were acquired using Multiclamp 700A (Axon Instruments). Signals were filtered at 1–2 kHz. All recordings under these protocols were digitized at 5 kHz and analyzed using pCLAMP 10 software (Axon Instruments). Experiments with a holding current of more than −100 pA or in which there was a change in input resistance >30% of the control were rejected. Whole-cell recordings were carried out with K-gluconate-based internal solution containing (in mM) 140 K-gluconate, 7 NaCl, 10 HEPES, 4 MgATP and 0.3 Na_3_-GTP, in which the pH was adjusted to 7.2 with KOH (OSM = 289).

### Microarray data analysis

Gene expression profile was analyzed using Illumina Human HT-12 v4.0 Expression BeadChip™ containing 47,306 probes. Raw data preprocessing for the Illumina beadchip microarray, including variance stabilizing transform and quantile normalization (for all 18 samples), was performed by the lumi package (Illumina) of the R Statistical package^30, 31^. Filtering transcripts with detection p-values < 0.05 in at least 50% of samples returned 20,213 probes for the following analysis. Probes associated with the same gene were summarized at the gene level expression by calculating median expression levels of the probes. Finally, we obtained expression profiles of 12,269 genes for each sample.

### Differential gene expression analysis

An empirical Bayes moderated t-statistics using the limma R package was used to determine differentially expressed genes in iNSC, iGN and ReNcell groups compared to control hADSC samples after correcting for multiple hypothesis testing by the Bonferroni method (p < 0.05), for which the absolute cutoff value of log fold change equaled 1.0^32^.

Heatmap representation of the genes that was identified by Kruskal-Wallis test followed by multiple comparison tests according to Conover p-value < 0.005. Heatmap was generated using the function heatplot in the ‘made4’ package of the Bioconductor project^33^. The distance between genes was calculated by 1–Pearson correlation, and subsequent clustering using the average method for two-dimensional hierarchical clustering.

### Functional annotation and pathway enrichment analysis

Differentially expressed genes were annotated with GO and Reactome pathways with the following enrichment analyses^34, 35^ using RDAVIDWebService (Benjamini-Hochberg FDR < 0.05 after one-tailed Fisher’s exact tests)^36^.

### Statistical analysis

All quantitative data were presented as mean ± standard error of mean (SEM) and evaluated using one-way analysis of variance (ANOVA) with Newman-Keuls *post hoc*. Statistical analysis was performed using the language R (R Development Core Team 2010). Probability (P)-values < 0.05 were considered statistically significant.

## Electronic supplementary material

Supplementary Information
