# Supplementary material for: Small molecule-based lineage switch of human adipose-derived stem cells into neural stem cells and functional GABAergic neurons
Source: Sci Rep. 2017 Aug 31;7:10166. doi: 10.1038/s41598-017-10394-y (PMC5579051; doi:10.1038/s41598-017-10394-y)
Supplement: Supplementary file 1 — Supplementary Information [file 41598_2017_10394_MOESM1_ESM.pdf]

Small molecule-based lineage switch of human adipose-derived stem cells into neural stem cells and functional GABAergic neurons

Jihye Park[1], Nayeon Lee[1], Jaekwang Lee[2], Eun Kyung Choe[3], Min Kyung Kim[4], Jeonghoon Lee[5], Min Soo Byun[6], Myong-Wuk Chon[7], Seong Who Kim[4], C. Justin Lee[2], Ju Han Kim[5], Jun Soo Kwon[6,8], Mi-Sook Chang[1,9]*

Supplementary Table S1. FACS analysis of hADSCs

		**hADSC 1**	**hADSC 2**	**hADSC 3**
**Positive markers**	CD73	98.83 %	99.74 %	99.21 %
	CD90	99.98 %	100 %	99.92 %
	CD105	99.63 %	99.79 %	98.89 %
	HLA-ABC	97.03 %	99.11 %	94.06 %
**Negative markers**	CD34	0.47 %	1.06 %	0.35 %
	CD45	0.03 %	0.14 %	0.39 %
	HLR-DR	0.03 %	0.10 %	0.44 %

Supplementary Table S2. Real-time PCR primer pairs

Gene name	Forward primer sequences	Reverse primer sequence	Product size (bp)	Gene Bank Accession
Ascl1	TGCACTCCAATCATTCACG	GTGCGTGTTAGAGGTGATGG	146	NM_004316
CALB2	CTCCAGGAATACACCCAAA	CAGCTCATGCTCGTCAATGT	207	NM_001740
Dlx2	GCACATGGGTTCCTACCAGT	TCCTTCTCAGGCTCGTTGTT	153	NM_004405
Dlx5	CCAACCAGCCAGAGAAAGAA	GCAAGGCGAGGTACTGAGTC	150	NM_005221
Emx1	AAGCGCGGCTTTACCATAGAG	GCTGGGGTGAGGGTAGTTG	150	NM_004097
FoxG1	AGAAGAACGGCAAGTACGAGA	TGTTGAGGGACAGATTGTGGC	189	NM_005249
GABRA1	GGATTGGGAGAGCGTGTAACC	TGAAACGGGTCCGAAACTG	66	NM_001127647
GABRA2	GTTCAAGCTGAATGCCCAAT	ACCTAGAGCCATCAGGAGCA	160	NM_000807
GABRA5	ATCTTGGATGGGCTCTTGG	TGTACTCCATTTCCGTGTCG	130	NM_000810
GAD65	GGTGGCTCCAGTGATTAAAG	TGTCCAAGGCGTTCTATTTC	165	NM_001134366
GAD67	AGGCAATCCTCCAAGAACC	TGAAAGTCCAGCACCTTGG	218	NM_000817
GAPDH	GTCAGTGGTGGACCTGACCT	CACCACCCTGTTGCTGTAGC	256	NM_001256799
GFAP	CAACCTGCAGATTCGAGAAA	GTCCTGCCTCACATCACATC	153	NM_002055
Gli3	TGGTTACATGGAGCCCCACTA	GAATCGGAGATGGATCGTAATGG	116	NM_000168
Lhx6	GGGCGCGTCATAAAAAGCAC	TGAACGGGGTGTAGTGGATG	108	NM_001242335
MAP2	CGCTCAGACACCCTTCAGATAAC	AAATCATCCTCGATGGTCACAAC	122	NM_002374
Musashi-1	TTCGGGTTTGTCACGTTTGAG	GGCCTGTATAACTCCGGCTG	250	NM_002442
Nestin	CACCTGTGCCAGCCTTTCTTA	TTTCCTCCCACCCTGTGTCT	170	NM_006617
Nkx2.1	GTGAGCAAGAACATGGCCC	AACCAGATCTTGACCTGCGT	182	NM_003317
Olig2	GCTGCGACGACTATCTTCCC	GCCTCCTAGCTTGTCCCCA	244	NM_005806
Pax6	AGGTATTACGAGACTGGCTCC	TCCCGCTTATACTGGGCTATTT	104	NM_001604
PVALB	GCTGAACGCTGAGGACATCAA	TCACATCATCCGCACTCTTTTTC	116	NM_002854
SCN1A	TGTCTCGGCATTGAGAACATTC	ATTGGTGGGAGGCCATTGTAT	220	NM_001202435
SCN5A	GGATCGAGACCATGTGGGAC	GCTGTGAGGTTGTCTGCACT	151	NM_001160161
Sox1	AGATGCACAACTCGGAGATCAG	GAGTACTTGTCCTTCTTGAGCAGC	184	NM_005986
Sox2	AGTCTCCAAGCGACGAAAAA	GCAAGAAGCCTCTCCTTGAA	141	NM_003106
SST	GCTGCTGTCTGAACCCAAC	CGTTCTCGGGGTGCCATAG	138	NM_001048
Tuj1	GGCCTTTGGACATCTCTTCA	ATACTCCTCACGCACCTTGC	241	NM_006086
vGat	CCGAGTGGTGAACGTAGCG	GTGGCGATAATGGACCAGGAC	135	NM_080552
vGlut2	GGGAGACAATCGAGCTGACG	CAGCGGATACCGAAGGAGATG	154	NM_020346
Vimentin	AGAACTTTGCCGTTGAAGCTG	CCAGAGGGAGTGAATCCAGATTA	255	NM_003380

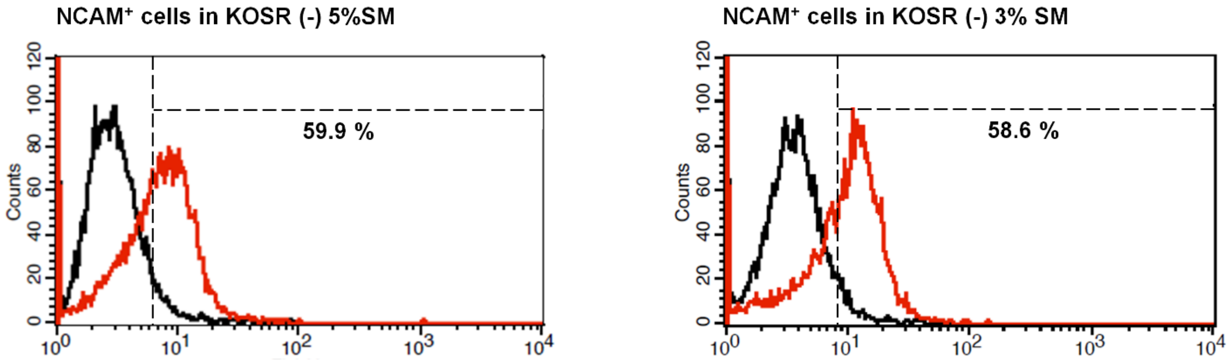

Supplementary Figure S1. Comparative analysis of NSC induction from hADSCs using different concentrations of knock-out serum replacement (KOSR). The efficiency of NSC induction protocols using different concentrations of KOSR (5% and 3%) was investigated by detecting the proportion of neural cell adhesion molecule (NCAM)-positive cells by flow cytometry.

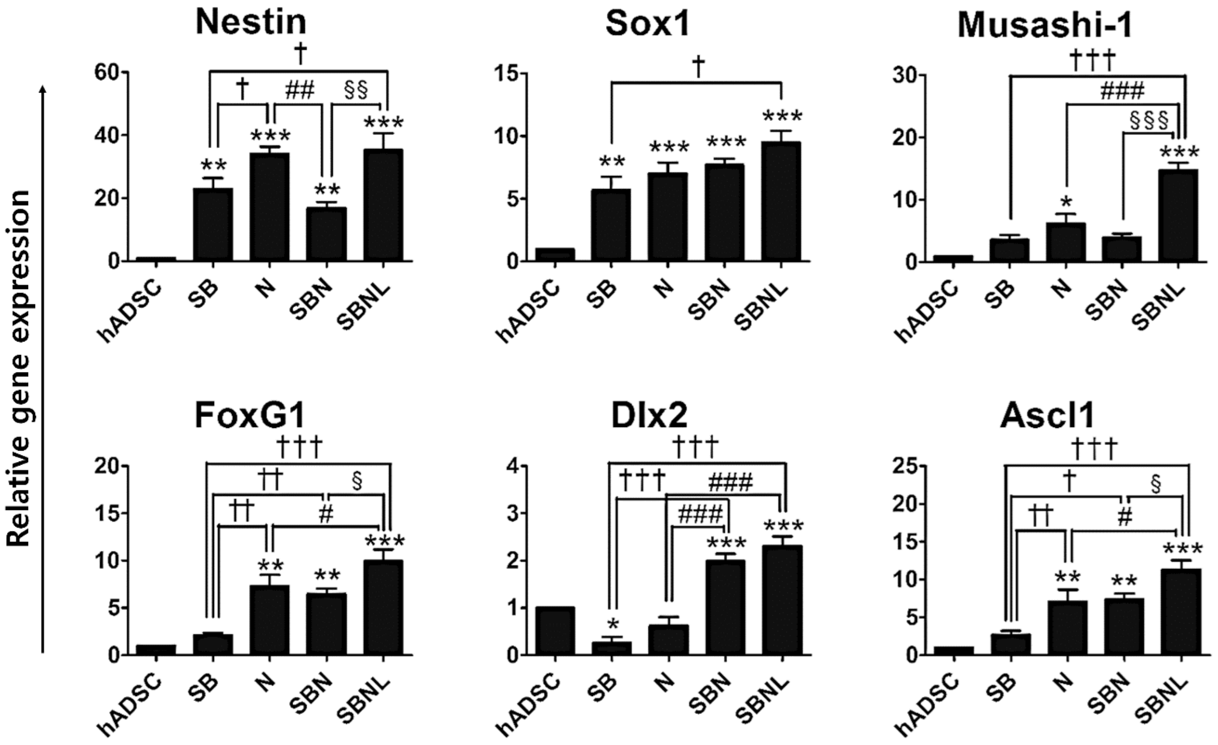

Supplementary Figure S2. Comparative analysis of NSC induction from hADSCs using different combinations of small molecules (SMs). The NSC marker expressions of cells induced from hADSCs using different combinations of the SMs, SB (SB431543), N (noggin), SBN (SB+N), and SBNL (SB+N+LDN193189), were investigated by real-time qPCR. *Nestin*, *Sox1*, *Musashi-1*, *FoxG1*, and *Ascl1* were significantly increased in SBNL conditions. *P < 0.05, **P < 0.01, ***P < 0.001 compared to hADSC, †P < 0.05, ††P < 0.01, †††P < 0.001 compared to SB, $^{\#}$P < 0.05, $^{\#\#}$P < 0.01, $^{\#\#\#}$P < 0.001 compared to N, §P < 0.05, §§P < 0.01, §§§P < 0.001 compared to SBN. ANOVA followed by post hoc Newman-Keuls test.

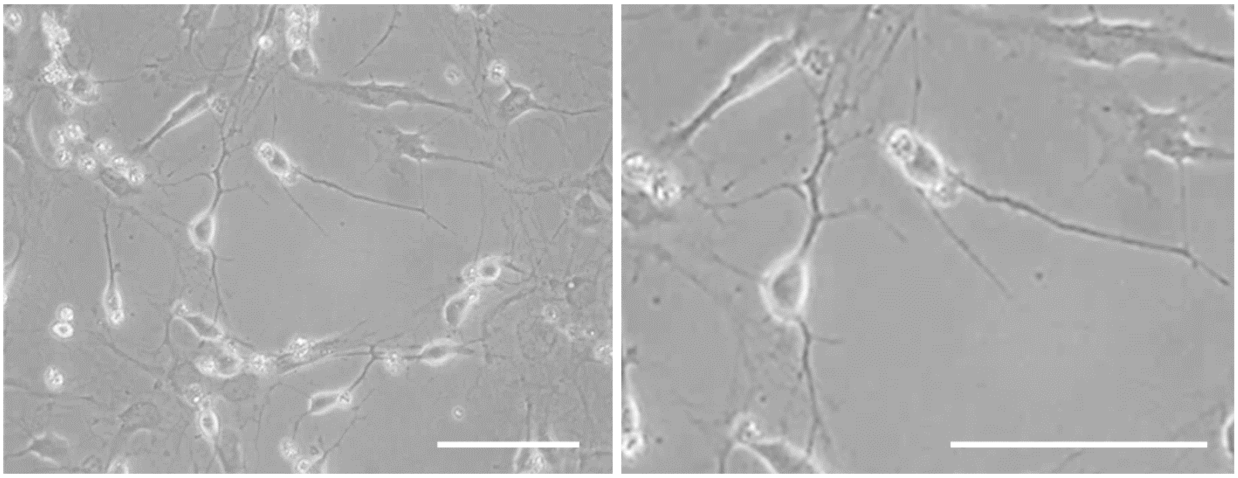

Supplementary Figure S3. Molphological feature of neuron-like cells (iNs) derived from hADSCs. The iNs displayed mature neuron-like morphology with distinct bipolar or multipolar neurites growing from the cell body. Scale bar: 100 μm.
